# Monthly Meteorological and Pathological Report of the City of Buffalo, for January 1847

**Published:** 1847-03

**Authors:** H. M. Congar


ART. IV. *Monthly Meteorological and Pathological Report of the City of Buffalo, for January* 1847. By H. M. Congar, M. D.

The instruments used in taking the observations, forming the body of the meteorological department of this report, are those united in the common Barometer, viz: a Hygrometer, a Spirit thermometer and a Barometer with a common Rain Gage, a Flag and a pocket Compass. The flag is fastened to a pole and fixed to the ridge of the roof of one of our highest buildings, by the side of it the rain-gage; the instrument containing the Barometer, Spirit thermometer, and Hygrometer, is hung in a box, so minutely perforated with five-eight of an inch holes on the sides as to secure complete atmospheric influence and yet so as not to admit rain, which is placed on the outside of a window in the third story of a high building; the instrument from the building is about six inches; there is also a stratum of air of an inch or more in thickness between the building and the box; air also enters freely at the bottom of the box. The times of taking the observations are those which can be most likely to be at the command of the observer and only twice in twenty-four hours at an equal distance from each other. During the month three of these observations were missed.

The number of Physicians in this city is 26. The whole number of cases of disease prescribed by them, as obtained from them by the reporter is 1370, about sixty of which were sent in without dates and these are not of course noticed in the daily enumerations. It must be borne in mind, that complete accuracy can not be obtained in this communication, from the difficulty on the part of the Physicians of remembering cases of single prescriptions and of charity patients because they were not at the time of occurence recorded. Yet this cause of inaccuracy, which at present is unavoidable, will we trust, as the habit of reporting is formed, in the future be entirely overcome, although it may be but *gradually* effected. We have connected as will be seen, the daily meteorological with the daily pathological phenomena.

The daily average number of cases occurring during the month is 42 6-31. The highest number is 78. The lowest number is 21. The number is below the average during fifteen days and above the average during sixteen days. Above during the prevalence of north-east and south-west winds and below during the prevalence of west and east wind. There were 312 cases of disease affecting the respiratory apparatus. 296 affecting the genito-urinary apparatus. 265 affecting the digestive apparatus. 142 affecting the cutaneous system. 119 affecting the blood and circulatory apparatus. 106 affecting the nervous apparatus. 89 affecting the solids or motive apparatus. And 30 affecting the optic apparatus.*

### CATALOGUE OF DISEASES.

*January* 1.—Pneumonia, 8. Cynanche, 6. Catarrh and Erysipelas, each 4. Accouchments, Derangements from Gestation and Dysentery, each 3. Peripneumonia Notha, Diarrhœa, Bronchitis, Anæmia, Gastric Derangements, Wounds, Gonorrhœa, Variola, and Asthma, each 2. Neuralgia, Dysuria, Remittent fever, Enteritis, Ephemeral fever, Dyspepsia, Intermittent fever, Orchitis, Scrofula, Phthisis, Leucorrhœ, Delirum tremens, Menorrhagia, Bilious Colic, Continued fever, Tinea capitis, Dysmenorrhœa, Ecchymosis, Epistaxis, Ulcerated sore throat, Metrorrhagia, Phrenitis, Opthalmia Hemorrhage from the bowels. Psoas abscess, Inflamed Breast, Schirrus, Scarlatina and Salt rheum, each 1.

* See table on next page.

### METEOROLOGICAL TABLE.

| January 1847. | Time. | Wind and Cloud. | Rain and Snow. | Hygrometer. | Thermom. | Barom. | No. Cases. |
|---|---|---|---|---|---|---|---|
| | a. m. | N E Strata, | 80-100 | 26 damp. | 42 | 29.51 | |
| 1 | 8½ p. m. | " " | 70-100 | 31 " | 36 | 29.33 | 78 |
| | a. m. | S W Cumulus. | | 37 " | 38 | 29.65 | |
| 2 | 8½ p. m. | " " | | 37 " | 36 | 29.73 | 65 |
| | a. m. | W " | | 30 " | 36 | 29.06 | |
| 3 | 8½ p. m. | W Clear. | | 30 " | 32 | 30.03 | 52 |
| | a. m. | E Cumulus. | R. but q'ty. | 25 " | 34 | 29.80 | |
| 4 | 8½ p. m. | S W strata. | Unknown. | 28 " | 42 | 28.90 | 46 |
| | a. m. | " cumulns. | | 25 " | 38 | 29.13 | |
| 5 | 8½ . m. | " " | | 23 " | 39½ | 29.35 | 40 |
| | a. m. | W clear. | | 21 " | 36 | 29.61 | |
| 6 | 8½ p. m. | N E " | | 21 " | 34 | 29.36 | 39 |
| | a. m. | " strata. | Snow. | 27 " | 38 | 28.89 | |
| 7 | 8½ p. m. | W cumulus. | | 20 " | 26 | 29.03 | 40 |
| | a. m. | " strata. | Snow. | 23 " | 19 | 29.49 | |
| 8 | 8½ p. m. | " cumulus. | | 27 " | 19 | 29.80 | 21 |
| | a. m. | S S W strata. | Snow. | 22 " | 24 | 29.88 | |
| 9 | 8½ p. m. | S W cumulus. | " | 33 " | 28 | 29.95 | 42 |
| | a. m. | W " | | 22 " | 18 | 29.97 | |
| 10 | 8½ p. m. | E strata. | Snow. | 21 " | 24 | 29.91 | 38 |
| | a. m. | W S W cumulus. | | 21 " | 20 | 29.81 | |
| 11 | 8¼ p. m. | W cumulus. | | 23 " | 19½ | 29.74 | 46 |
| | a. m. | S W " | | 20 " | 19 | 30.06 | |
| 12 | 8½ p. m. | W " | | 22 " | 23 | 30.17 | 46 |
| | a. m. | S W " | | 17 " | 31 | 29.96 | |
| 13 | 8½ p. m. | " clear. | | 20 " | 36 | 29.68 | 43 |
| | a. m. | " strata. | | 27 " | 39 | 29.59 | |
| 14 | 8½ p. m. | | | 27 " | 40 | 29.54 | 38 |
| | a. m. | S E strata. | | 30 " | 46 | 29.30 | |
| 15 | 8½ p. m. | S W " | Rain. | 29 " | 50 | 29.13 | 43 |
| | a. m. | " cumulus. | " 6-10 | 32 " | 40 | 29.26 | |
| 16 | 8½ p. m. | W clear. | | 15 " | 19 | 30.04 | 31 |
| | a. m. | E cumulus. | | 17 " | 19 | 30.19 | |
| 17 | 8½ p. m. | | | 15 " | 22 | 30.02 | 32 |
| | a. m. | S W strata. | | 16 " | 35 | 29.49 | |
| 18 | 8½ p. m. | W | | 24 " | 32 | 29.39 | 52 |
| | a. m. | " cumulus. | | 17 " | 18 | 29 72 | |
| 19 | 8½ p. m. | " " | | 14 " | 15 | 30.05 | 41 |
| | a. m. | S W " | | 15½ " | 16 | 30.21 | |
| 20 | 8½ p. m. | W strata. | Snow. | 18 " | 23 | 30.11 | 36 |
| | a. m. | " cum. strat. | | 16 " | 20 | 29.94 | |
| 21 | 8½ p. m. | " " " | | 15 " | 12 | 29.98 | 44 |
| | a. m. | " cumulus. | | 17 " | 12 | 30.02 | |
| 22 | 8½ p. m. | " stratus. | | 16 " | 20 | 29.67 | 33 |
| | a. m. | S W cir. cum. | | 18 " | 28 | 29.39 | |
| 23 | 8½ p. m. | " clear. | | 20 " | 32 | 29.47 | 38 |
| | a. m. | " cumulus. | | 23 " | 30 | 29.52 | |
| 24 | 8½ p. m. | W clear. | | 20 " | 28 | 29.83 | 28 |
| | a. m. | N E cir. cum. | | 20 " | 21 | 30.00 | |
| 25 | 8½ p. m. | " " " | | 19 " | 30 | 29.95 | 44 |
| | a. m. | S W stratus. | Rain. | 27 " | 38 | 29.37 | |
| 26 | 8½ p. m. | W cumulus. | 4-10 inch. | 27 " | 30 | 29.62 | 48 |
| | a. m. | N E " | | 23 " | 24 | 29.96 | |
| 27 | 8½ p. m. | " clear. | | 21 " | 19 | 30.27 | 41 |
| | a. m. | " " | | 22 " | 16 | 30.36 | |
| 28 | 8½ p. m. | " cir. cum. | | 21 " | 29 | 30.00 | 42 |
| | a. m. | E " " | | 19 " | 33 | 29.68 | |
| 29 | 8½ p. m. | N E cir. stratus | Sleet. | 29 " | 36 | 28.96 | 45 |
| | a. m. | N W stratus. | Snow. | 30 " | 26 | 29.12 | |
| 30 | 8½ p. m. | cumulus. | | 22 " | 22 | 29.40 | 39 |
| | a. m. | S clear. | | 23 " | 22 | 29.47 | |
| 31 | 8½ p. m. | S W cumulus. | | 23 " | 27 | 29.39 | 37 |
| Wind W. 10 days. | | Clear, 5 days. | Rain in 5 days, 2½ inches. | 37 damp H. | 50 H. | 30.36 H. | |
| " S.W. 9½ " | | Stratus, 7½ " | | 22 3-5 " M. | 28 12-31 M | 29.80 M. | |
| " N. E. 5½ " | | Cum., 13 " | Snow, 6 days. | 14 " L. | 12 L. | 28.89 L. | |
| " E. 2 " | | Cum. str., 1 " | Sleet, 1 day. | 23 " R. | 38 R. | 1.57 R. | |
| " S. E. ½ day. | | Cir. cum., 2½ " | | | | | |
| " S.S.W. ¼ " | | Unknown, 1½ " | | | | | |
| " W.S.W. ½ " | | | | | | | |
| " N. W. ½ " | | | | | | | |
| " S. ⅛ " | | | | | | | |
| " unknown, 1½ | | | | | | | |

2.—Pneumonia and Conjunctivitis, each 4. Accouchments, Neuralgia, Phthisis, Derangements from Gestation, Gastric derangements. Gonorrhœa and Variola, each 3. Peripneumonia Notha, Infantile convulsions, Rheumatism, Ecchymosis, Chronic ulcerative Pharyngitis and Scrofula, each 2. Catarrah, Dysuria, Remittent Fever, Diarrhœa, Bronchitis, Dyspepsia. Pertussis, Intermittent Fever, Orchitis, Wounds, Laryngitis, Tape Worm, Spinal Irritation, Continued Fever, Scarlatina, Croup, Uterine Hemorrhoge, Fractured cranium, Poisoning by oil of tanzy, Pleurisy, Congestion of the Brain, Hysteritis, Necrosis, Erysipelas, Fungus Hæmatides with removal of the whole eye, and Otorrhœa with Crustæ Lactæ, each 1.

3.—Accouchments, 4. Bilious Colic and Spinal Irritation, each 3. Catarrh, Neuralgia, Peripneumonia Notha, Hydrocephalus and Derangements from Dentition, each 2. Abortion, Hepatitis, Enteritis, Diarrhœa, Ephemeral Fever, Bronchitis, Dyspepsia, Orchitis, Scrofula, Phthisis, Derangements from Gestation, Ulcerated Tonsilitis, Infantile convulsions, Gastric derangements, Ulcerated Cornea, Pneumonia, Chronic Pneumonia, Hysteria, Worms, Conjunctivitis, Impotence, Prolapsus Uteri, Fractured Clavicle, Vaccination, Syphilis, Dysmenorrhœa, Scarlatina, Influenza and Inflamed Eye, each 1.

4.—Accouchments, Abortions and Catarrhs, each 3. Intermittent Fever, Scrofula, Phthisis, Gonorrhœa, Vaccination, Burn and Pleurisy, each 2. Neuralgia, Dropsy, Diarrhœa, Bronchitis, Orchitis, Piles, Measles, Continued Fever, Porrigo, Pneumonia, Phrenitis, Sprained Ankle, Paralysis, Syphilis, Derangements from Dentition, Lead Colic, False Conception, Nervous Headache, Cough, Chronic Pharyngitis and Impetigo, each 1.

5.—Vaccination, 4. Diarrhœa and Scrofula, each 3. Ephemeral Fever, Bronchitis, Gastric derangements and Tinea Capitis, each 2. Accouchment, Catarrh, Abortion, Neuralgia, Remittent Fever, Enteritis, Dropsy, Dyspepsia, Anæmia, Phthisis, Leucorrhœa, Gonorrhœa, Menorrhagia, Rheumatism, Uterine Hemorrhage thirty-four days after Labor, Suppression of the Menses, Prolapsus Uteri, Ulcer, Burn, Phrenitis, Spermatorrhœa and Drowning, each 1.

6.—Catarrh, 4. Scrofula, 3. Accouchments, Hepatitis, Croup, Pneumonia and Syphilis, each 2. Abortion, Varicella, Remittent Fever, Diarrhœa, Dyspepsia, Intermittent Fever, Chronic Pharyngitis, Constipation, Phthisis, Gastritis, Gastric derangement, Gonorrhœa, Dysentery, Continued Fever, Vaccination, Herpes Circinnatus, Pleurisy, Hydrocephalus, Nephritis, Bronchocele, Diseased valves of the Heart and Phyma Anthrax, each 1.

7.—Accouchments and Gastric derangements, each 4. Diarrhœa, 3. Dyspepsia, Phthisis and Pneumonia, each 2. Abscess, Abortion, Varicella, Bronchitis, Anæmia, Chronic Pharyngitis, Scrofula, Constipation, Piles, Derangement from Gestation, Gonorrhœa, Dysentery, Rheumatism, Croup, Prolapsus Uteri, Syphilis, Hydrocephalus, Malis Pediculi, Paralysis of the neck of the Bladder and Inflamed Knee, each 1.

8.—Pneumonia and Syphilis, each 3. Accouchments, Catarrh, Neuralgia, Dysmenorrhœa and sore Nipples, each 2. Phthisis, Wound, Gonorrhœa, Laryngitis and Worms, each 1.

9.—Catarrh, 4. Bronchitis, 3. Gonorrhœa, Continued Fever, and Marasmus, each 2. Accouchments, Hepatitis, Enteritis, Dropsy, Diarrhœa, Pneumonia, Ephemeral Fever, Pertussis, Intermittent Fever, Scrofula, Piles, Coughs, Leucorrhœa, Derangement from Gestation, Wounds, Laryngitis, Dysentery, Croup, Hysteria, Worms, Aphthæ, Dislocated Humerus, Periostitis, Pleurisy Cynanche, Pruritus, Threatened Abortion, Inflamed knee and Bright's Disease, each 1.

10.—Accouchments, 7. Pneumonia, 4 Scarlatina, 3. Catarrh, Abortion, Derangement from Gestation, Gonorrhœa, Continued Fever, and Syphilis, each 2. Dropsy, Constipation, Gastric derangement, Spinal Curvature, Spinal Irritation, Hysteria, Jaundice, Suppression of the Menses, Vaccinations, Fractured Finger, Hæmiplagia and Erysipilas, each 1.

11.—Cough, 4. Catarrh, Abortions, and Gastric derangement, each 3. Acouchments, Diarrhœa, Intermittent Fever, Leucorrhœa, Pneumonia, and Ulcerated Sore Throat, each 2. Abscess, Hepatitis, Enteritis, Dyspepsia, Anæmia, Piles, Tonsilitis, Gonorrhœa, Continued Fever, Porrigo, Typhoid Pneumonia, Retention of the Menses, Ulcers, Syphilis, Poisoning by Opium, Fractured Femur,

Epilepsy, Masturbation, Influenza, Inflamed breast and Dysuria, each 1.

12. Accouchments, 6. Diarrhœa, 3. Enteritis, Bronchitis, Gastric derangement, Measles and Vaccination, each 2, Abscess Catarrh, Abortion, Hepatitis, Remittent Fever, Ephemrral Fever, Intermittent Fever, Anæmia, Scrofula, Derangement from Gestation, Tonsilitis, Infantile Convulsions, Gastro Enteritis, Gonorrhœa, Laryngitis, Spinal Irritation, Pneumonia, Ulcerative Sore Mouth, Intestinal Irritation, Prolapsus Uteri, Pleurisy, Fractured Femur, Ulcerated Soie Throat, Parotitis, Erysipilas, Cholera Morbus, and Impetigo, each 1.

13.—Accouchments, Gastric Derangements and Cough, each 4. Infantile Convulsions, Croup, Hysteria, Dysmenorrhœa and Pruritis, each 2. Infantile Marasmus, Hepatitis, Ephemeral Fever, Dyspepsia, Intermittent Fever, Anasarca, Scrofula, Phthisis, Derangement from Gestation, Spinal Irritaiion, Continued Fever, Pneumonia, Syphilis, Poisoning from Corrosive Sublimate, Tinea Capitis, Phrenitis, Excised Tonsils, Comminuted fracture of Tibea and Fibula, Diabetes Insipidus, Dysura and Eczema, each 1.

14.—Catarrh, 4. Abscess, 3. Accouchment, Ephemeral Fever, Intermittent Fever, Anæmia, Derangement from Gestation and Pneumonia, each 2. Abortion, Neuralgia, Hepatitis, Enteritis, Dyspepsia, Constipation, Purulent Ophthalmia, Gastric derangement, Gastritis, Gonorrhœa, Delirium Tremens, Rheumatism, Amenorrhœa, Syphilis, Dysmenorrhœa, Pleurisy, Fractured Femur, Nasal Polypus and Abdominal Tumor, each 1.

15.—Accouchments, 6. Pneumonia, 4. Catarrh and Dyspepsia, each 3. Hepatitis, Bronchitis, Epilepsy and Inflamed Breast, each 2. Enteritis, Dropsy, Diarrhœa, Ephemeral Fever, Ophthalmia, Gastric derangement, Wound, Gonorrhœa, Croup, Hysteria, Vaccination, Coxalgia, Hæmiplegia, Gleet, Hysteritis, Scarlatina, Inflamed Synovial Membrane of the Knee, Vescico-Vaginal Fistula and Needle in the Knee, each 1.

16.—Phthisis, 4. Accouchments, 3. Catarrh, Bronchitis, Dyspepsia, Spinal Irritation, Bruised Scalp and Vaccination, each 2. Abscess, Acute Hepatitis, Chronic Hepatitis, Dropsy, Diarrhœa, Ephemeral Fever, Derangement from Gestation, Leucorrhœa, Infantile Convulsions, Measles, Dysentery, Croup, Aphthæ, Conjunctivitis, Variola, Sore Throat, Masturbation, Hysteritis, Hæmatemesis and Periodical Headache, each 1.

17.—Catarrh and Gonorrhœa, each 3. Bronchitis, Piles, Vaccination and Erysipelas, each 2. Accouchments, Neuralgia, Varicella, Hepatitis, Diarrhœa, Derangement from Gestation, Gastric derangement, Laryngitis, Croup, Pneumonia, Sprained Ankle, Syphilis, Tumor, Pleurisy, Epistaxis, Phrenitis, Hæmatemesis and Inflamed Synovial Membrane of the Knee, each 1.

18.—Vaccinations, 9. Accouchments, 4. Gastric derangement and Croup, each 3. Catarrh, Dropsy, Tonsilitis, Pneumonia and Amenorrhœa, each 2. Infantile Marasmus, Neuralgia, Varicella, Hepatitis, Remittent Fever, Diarrhœa, Ephemeral Fever, Bronchitis, Phthisis, Infantile Convulsions, Dysentery, Chronic Pneumonia, Coma, Sprained Ankle, Prolapsus Ani, Ulcer, Gastralgia, Tinea Capitis, Hydrocephalus, Sore Throut, Threatened Abortion, Foreign body in the Esophagus and Scarlatina, each 1.

19.—Phthisis and Pneumonia, each 4. Catarrh, 3. Hepatitis, Bronchitis, Dyspepsia and Gleet, each 2. Abscess, Accouchment, Infantile Marasmus, Neuralgia, Dropsy, Diarrhœa, Ephemeral Fever, Derangement from Gestation, Leucorrhœa, Infantile Convulsions, Arachnitis, Spinal irritation, Typhoid Continued Fever, Croup, Psoriasis, Paralysis, Dysmenorrhœa, Epilepsy, Inflamed Knee, Peritonitis and Excised Uvula, each 1.

20.—Gastric derangement, 4. Pneumonia, 3. Catarrh, Gonorrhœa, Dysuria, Croup and Ulcerated sore Throat, each 2. Accouchment, Neuralgia, Hepatitis, Remittent Fever, Dyspepsia Intermittent Fever, Infantile Convulsions, Pleuralgia, Ulcerated Cornea, Continued Fever, Scarlatina, Worms, Conjunctivitis, Syphilis, Burn, Gastralgia, Phrenitis, Influenza and Cough, each 1.

21.—Vaccination, 10. Catarrh and Pneumonia, each 3. Abscess, Accouchment, Neuralgia, Chronic Hepatitis, Enteritis, Diarrhœa, Derangement from Gestation, Intermittent Fever, Leucorrhœa, Tonsilitis, Infantile Convulsions, Gastric derangement, Gonorrhœa, Menorrhagia, Bilious Colic, Spinal Irritation, Rheumatism, Continued Fever, Hysteria, Ulceration, Sore Mouth, Phrenitis,

Paralysis, Boil, Syphilis, Burn, Gastralgia, Epilepsy and Hysteritis, each 1.

22.—Accouchments, Pneumonia and Amenorrhœa, each 3. Catarrh, Abortion, Infantile Marasmus and Varicella, each 2. Neuralgia, Dyspepsia, Phthisis, Tonsilitis, Gastric derangement, Gonorrhœa, Gonorrhœal Ophthalmia, Croup, Hysteria, Palpitation from diseased Heart, Dislocated Ankle with fractured Tibia, Vaccination, Iritis, Ecchymosis, Pleurisy and Hydrocephalus, each 1.

23.—Catarrh, Neuralgia, Varicella and Vaccination, each 3. Dyspepsia, Scrofula, Bilious Colic, Rheumatism, Croup and Pneumonia, each 2. Diarrhœa, Derangement from Gestation, Phthisis, Gastric derangement, Wound, Gonorrhœa, Continued Fever, Sprained Ankle, Amenorrhœa, Prolapsus Ani, Boil, Chilblain, Nephritis and Cough, each 1.

24.—Measles, 4. Accouchment, Neuralgia, Gonorrhœa, Rheumatism, Continued Fever and Croup, each 2. Catarrh, Dysuria, Bronchitis, Wound, Arachnitis, Bilious Colic, Dysentery, Pneumonia, Periostitis, Erysipelas, Fractured Tibia and Influenza, each 1.

25.—Catarrh, Neuralgia, Bronchitis, Tonsilitis, Gastric Derangement, Rheumatism, and Pneumonia, each 2. Accouchment, Abortion, Dyspepsia, Pertussis, Anæmia, Scrofula, Leucorrhœa, Gonorrhœa, Roseola, Delirium Tremens, Ulcerated Cornea, Scarlatina, Croup, Aphtha, Salivation, Conjunctivitis, Prolapsus Uteri, Syphilis, Herpes Circinnatus, Burn, Poisoning from Opium, Tinea Capitis, Dysmenorrhœa, Pleurisy, Cynanche, Derangement from Dentition, Threatned Abortion, Herpes, Head-ache from Carbonic acid gas from Charcoal, and Puerpal Fever, each 1.

26.—Vaccinations, 5. Accouchments, Bronchitis, Constipation, Gonorrhœa and Influenza, each 3. Rheumatism, 2. Neuralgia, Hepatitis, Remittent Fever, Derangement from Gestation, Anæmia, Scrofula, Leucorrhœa, Gastric Derangement, Measles, Spinal Irritation, Continued Fever, Croup, Hysteria, Gangrene, Serous effusion in the Nymphæ, Scabies, Intestinal Irritation, Conjunctivitis, Fractured Clavicle, Burn, Gastralgia, Tinea Capitis, Pleurisy, Cynanche, Bruised Leg and Ankle, and Gastric Hernia, each 1.

27.—Catarrh, 4. Gastric derangement and Wound, each 3 Infantile Convulsions, Measles, Continued Fever and Syphilis, each

2. Accouchment, Hepatitis, Enteritis, Diarrhœa, Ephemeral Fever, Pertussis, Scrofula, Gonorrhœa, Scarlatina, Croup, Apoplexy Sprained Ankle, Scabies, Intestinal Irritation, Conjunctivitis, Vaccination, Poisoning by Corrosive Sublimate, Sick Headache, Pleurisy, Cynanche. Concussion, of the Brain, Echphlysis Phompholix, and Granulated Eye-lids with sloughing Cornea, each 1.

28.—Varicella and Pneumonia, each 3, Accouchment, Enteritis, Diarrhœa, Bronchitis, Constipation and Amenorrhœa, each 2. Catarrh, Neuralgia, Hepatitis, Intermittent Fever, Anæmia, Scrofula, Purulent Opthalmia, Gastric derangement. Wound, Menorrhagia, Laryngytis, Spinal Irritation, Pleuralgia, Scarlatina, Croup, Prolapsus Uteri, Burn, Epistaxis, Congestion of the Brain, Derangement from Dentition, Periodical Head ache, Dysphoria Simplex, Ovarian Tumor and Cough, each 1.

29.—Accouchments, 7. Syphilis *3. Gastric derangement, Spinal Irritation, Croup, Pneumonia, Influenza Cynanche and Scarlatina, each, 2. Catarrh, Neuralgia, Peripneumonia Notha, Dropsy, Diarrhœa, Ephemeral Fever, Dyspepsia, Phthisis, Laryngitis, Bilous Colic, Pleuralgia, Sprained Ankle, Rachitis,Conjunctivitis, Vaccination, Dislocated Humerus, Irritable Uterus, Excessive Venery, Amaurosis and Cataract, Hæmorrhage from extraction of Tooth and Periodical Head-ache, each 1.

30.—Catarrh, 5. Varicella, Chronic Hepatis, Diarrhœa Pneumonia, Granulated Eye-lids, Influenza and Cough, each 2. Neuralgia, Hepatitis, Remittent Fever, Ephemeral Fever, Derangement from Gestation, Dyspepsia, Constipation, Leucorrhœa, Infantile Convulsions, Gastric Derangement, Wound, Laryngitis, Bilious Colic, Inflamed Breast, Croup, Jaundice, Ulcer, Headache with furious Delirum, Pleurisy, and Threatened Abortion, each 1.

31.—Accouchments, 6. Catarrh, 4. Dyspepsia and Croup, each. 2. Neuralgia, Varicella, Passage of Gall-stones, Remittent Fever, Chlorosis,Dropsy, Pneumonia, Wound, Gonorrhœa, Laryngitis, Rheumatism, Continued Fever. Uterine Hæmorrhage, Syphilis, Sick Head-ache, Amaurosis and Cataract, Hæmorrhage from the Bowels, Gout, Periodical Head-ache, UlceratedColon, Scarlatina, Ectropia, and Granulated, Eye-lids, each 1.

*Total for January.*—Accouchments, 85. Catarrhs, 73. Pneumonia, 70. Gastric Derangements, 51. Vaccination, 47. Gonorrhœa, 37. Croup, 33. Bronchitis, 34. Diarrhœa, 30. Neuralgia, 29. Dyspepsia, 27. Hepatitis and Scrofula, each 26. Influenza and Derangement from Gestation, each 22. Continued Fever and Phthisis, each 21. Cynanche and Syphilis, each 20. Abortions, 19. Wounds, Ephemeral Fever and Rheumatism, each 18. Intermittent Fever, 16. Spinal Irritation, 15. Varicella and Infantile Convulsions, each 14. Pleurisy, 13. Enteritis, Cough and Tonsilitis, each 12. Dropsy, Leucorrhœa, Measles, Conjunctivitis, Erysipelas and Anæmia, each 11. Remittent Fever, Constipation, Hysteria and Amenorrhoea, each 10. Abscess, Laryngitis, Bilious Colic, Dysentery, Derangement from Dentition, Scarlatina and Dysmenorrhœa. each 9. Burns, 8. Dysuria, Peripneumonia Notha, Tinea Capitis and Phrenitis, each 7. Sprained Ankle, Variola, Gastralgia, Poisoning, Prolapsus Uteri, Epilepsy, Hydrocephalus and Piles, each 6. Infantile Marasmus, Delirium Tremens, Inflamed Breast, Scarlatina and Granulated Eyelids, each 5. Pertussis, Orchitis, Purulent Ophthalmia, Menorrhagia, Worms, Ulcer, Boil, Ecchymosis, Sick Headache, Periodical Headache, Hysteritis and Threatened Abortion, each 4. Ulcerated Cornea, Aphthæ, Uterine Hæmorrhage, Psoriasis, Pertussis, Prolapsus Ani, Periostitis, Tumor, Epistaxis, Fractured Femur, Pruritus, Hæmorrhage, Parotitis, Gleet and Inflamed Knee, each 3. Chronic Pharyngitis, Chlorosis, Gastritis, Arachnitis, Porrigo, Ulcerative sore Mouth, Intestinal Irritation, Jaundice, Suppression of the Menses, Bruised Scalp, Fractured Clavicle, Dislocated Humerus, Herpes Cincinnatus, Amaurosis, Asthma, Cerebral Congestion, Lead Colic, Nephritis, Hemiplegia, Hæmatemesis, Hemoptysis, Masturbation, Marasmus, Inflamed Synovial Membrane of the Knee, Pharyngitis and Impetigo, each 2. Roseola, Tape Worm, Spinal Curvature, Gonorrhœal Ophthalmia, Retention of the Menses, Palpitation, Gangrene, Coma. Dislocated Ankle with fractured Tibia, Apoplexy, Salivation, Serous effusion in the Nymphæ, Scabies, Rachitis, Impotence, Fractured Cranium, Chilblain, Iritis, Irritable Uterus, Excision Venery, Fractured Finger, Amaurosis and Cataract, Metrorhagia, Scrofulous Ophthalmia, Psoas Abscess, Spermatorrhœa, Schirrus, Hydrocele, Coxalgia, Concussion of the Brain, Gout, Drowning, Ecphlysis Pompholix, Necrosis, Dysphoria Simplex, Ulcerated Colon, Fractured Tibia, Fungus Hematodes with removal of the Eye, Excised Tonsils, Ectropia, Herpes, Bruised Leg and Ankle, Ovarian Tumor, Malis Pediculi, Sore Nipples, Cholera Morbus, Headache from Carbonic Acid gas from Charcoal, Salt Rheum, Valvular disease of the Heart, Paralysis of the neck of the Bladder, Comminuted fracture of the Leg, Nasal Polypus, Abdominal Tumor, Bright's Disease, Diabetes Insipidus, Peritonitis, Foreign body in the Esophagus, Excised Uvula, Gastric Hernia, False Conception, Nervous Headache, Puerperal Fever, Otorrhœa with Crusta Lactea, Eczema, Fistula, Needle in the Knee, Urticaria and Phymosis, each 1.

